# Response of future hydropower generation of cascade reservoirs to climate change in alpine regions

**DOI:** 10.1371/journal.pone.0269389

**Published:** 2022-08-19

**Authors:** Bing Yan, Yi Xu, Heng Liu, Changshuo Huang

**Affiliations:** 1 Hydrology and Water Resources Department, Nanjing Hydraulic Research Institute, Nanjing, China; 2 College of Hydrology and Water Resources, Hohai University, Nanjing, China; 3 State Key Laboratory of Hydrology Water Resources and Hydraulic Engineering, Nanjing, China; Universidade de Vigo, SPAIN

## Abstract

Climate warming accelerates the hydrological cycle, especially in high-latitude and high-altitude areas. The increase in temperature will increase the amount of snow and glacier melting and change the runoff, which will affect the operations of cascade reservoirs significantly. Therefore, taking the upper reaches of the Yellow River with an alpine climate as an example, we propose an improved SIMHYD-SNOW, which considers the snowmelt runoff process. The impacts of climate changes on the runoff process were revealed based on the SIMHYD-SNOW model using the precipitation and temperature data predicted by the SDSM model. A model for the maximum power generation of the cascade reservoirs in the upper reaches of the Yellow River was constructed to explore the impacts of climate changes on the inter-annual and intra-annual hydropower generation of the cascade reservoirs at different periods in the future. The results show that climate change has changed the spatial and temporal allocation of water resources in this area. The future runoff will decrease during the flood period (July to September) but increase significantly during the non-flood period. The inter-annual and intra-annual hydropower generation under the RCP8.5 climate change scenario is significantly lower than the RCP2.6 and RCP4.5 climate change scenarios, and as the CO_2_ emission concentration increases, this gap increases significantly. This study can provide technical references for the precise formulation of water resources management under climate change.

## 1. Introduction

In recent years, the response characteristics of reservoir operation results to climate change have attracted more and more attention, especially in high-latitude and high-altitude areas [1, 2]. The response of snow, glaciers and frozen soil to climate change is particularly prominent [3–6]. Climate change and intensive human activities have changed the temporal and spatial distribution of water resources in these areas, and significantly affect local hydropower generation. Reservoir regulation is an important measure to realize normal operation and redistribute water resources in time and space [7, 8]. Therefore, predicting the river runoff and hydropower generation process under the conditions of future climate change has scientific significance for deeply understanding the water resource evolution.

The methods to predict future runoff can be divided into statistical data-driven methods and physical hydrological models [9, 10]. The former use classical regression analysis, BP neural network, nonlinear time series analysis and fuzzy mathematical methods to establish a statistical relationship between meteorological and runoff elements so as to predict the future runoff [11, 12]. The latter is used to simulate the relationship between rainfall and runoff based on certain physical mechanisms which integrate the characteristics of the underlying surface and rainfall-runoff producing in the basin and have been widely used [13–16]. In addition, the Global Climate Model (GCM) is an important tool for understanding the mechanism of climate change and predicting future climate change. It simulates the main physical and chemical processes of the global atmosphere, ocean, land, and sea ice to predict future climate scenarios. In terms of climate change prediction, the precipitation and temperature data of 12 GCMs under RCP2.6 and RCP8.5 scenarios (representative concentration pathway, radiation forcing levels by 2100 are 2.6 W/m^2^ and 8.5 W/m^2^, respectively) were used to drive the Budyko model to predict the runoff changes of 37 tributaries in the Yellow River Basin from 2070 to 2099 [16]. A hybrid model for monthly runoff prediction based on the monthly inflow runoff data from two reservoirs in the Yangtze River Basin and found that the model provides better prediction accuracy than the traditional artificial neural network and the extreme learning machine method [17].

In terms of the response of climate change to the operation of reservoirs, the research of coupling hydrological model and reservoir operation model has attracted more and more attention [18, 19]. For example, the impact of climate change and land use change on the hydropower generation process by coupling GCM and SWAT models was explored [20]. A joint operation model of dry-flow cascade reservoirs with the Longyangxia, Liujiaxia, and Xiaolangdi cascade reservoirs as the core, and pointed out the operation characteristics of different reservoirs in the upper and lower reaches of the Yellow River during the flood season and the peak water consumption period [21].

The Yellow River is an important water source in Northwest and North China. Climate change has severely restricted the sustainable use of water resources and affected food production in the Yellow River Basin, which is an important rain-fed agricultural area in China [22]. The upper reach of the Yellow River is located in alpine region with an average annual runoff of about 24.872 billion m^3^, accounting for more than 40% of the whole river. It is an important runoff-producing area in the Yellow River Basin [22]. But with the climate warming, extreme floods and droughts have brought severe challenges to the operation and management of cascade reservoirs in the upper reaches of the Yellow River [23].

Therefore, this study takes the cascade reservoirs in the upper reaches of the Yellow River as examples, in order to explore the impact of climate change on the future operations of reservoirs in different periods. Firstly, the Mann-Kendall method was used to identify the runoff mutations from Tangnaihai and Xiaochuan hydrological stations. Then an improved SIMHYD-SNOW model was proposed by fully considering the snow melting process, and its parameters were calibrated by using the Particle Swarm Optimization (PSO) algorithm. Then, the statistical downscaling model (SDSM) was used to downscale the climate data of the three climate change scenarios (RCP2.6, RCP4.5 and RCP8.5) generated by four climate models (CanESM2, CNRM-CM5, MIROC5 and BNU-ESM) to obtain future meteorological elements, which were used as inputs by the SIMHYD-SNOW model to predict the future inflows to the Longyangxia (LYX) and Liujiaxia (LJX) reservoirs. Finally, the optimal dispatching model for maximum hydropower generation of the LYX and LJX cascade reservoirs on the upper reaches of the Yellow River was constructed, and the response mechanism to climate change was explored.

## 2. Study areas and data sources

**Fig 1:**
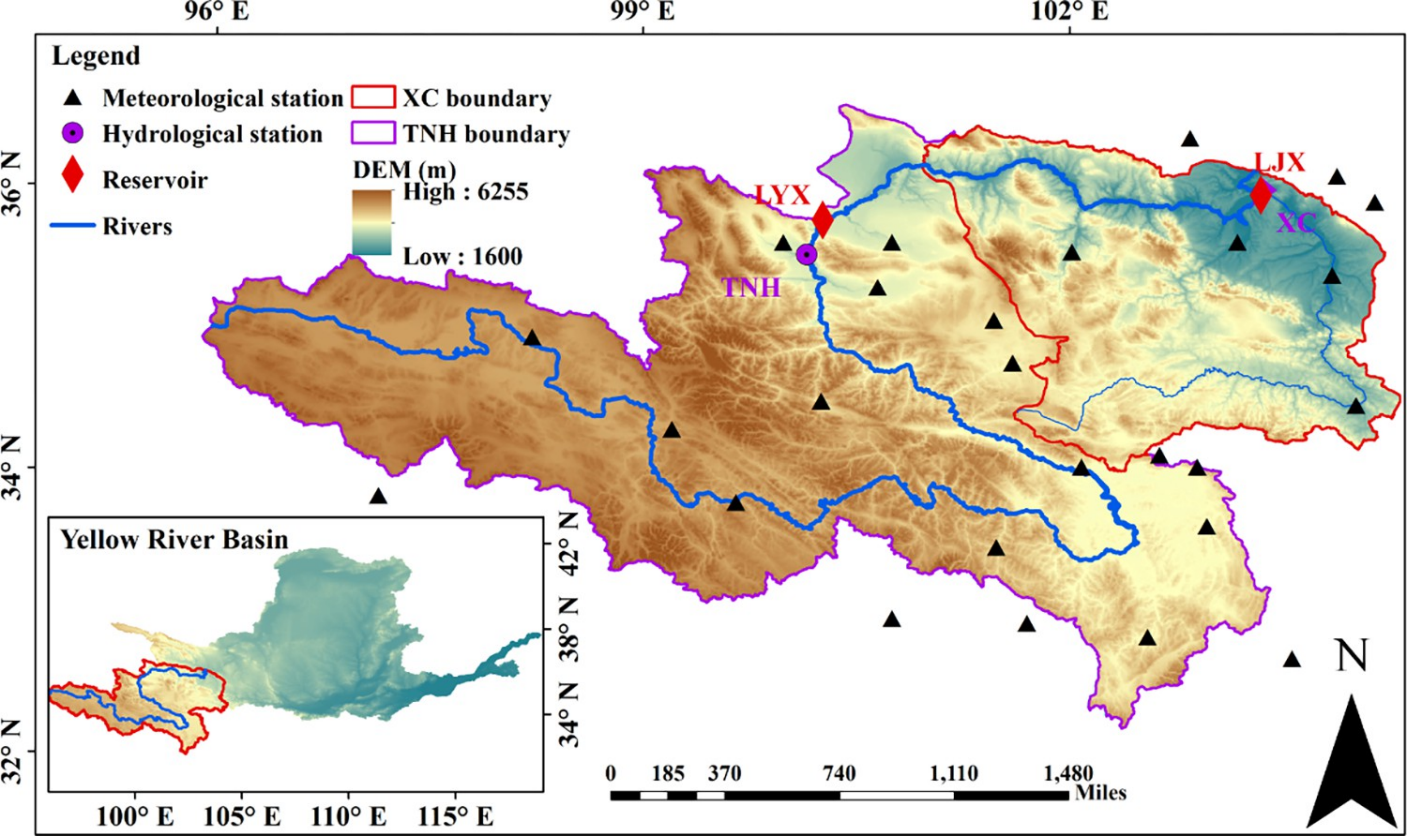
The geographical location and the spatial distribution of meteorological and hydrological stations.

The Yellow River Basin covers an area of 795,000 km^2^ with its mainstream length of 5464 kilometers, accounting for about 8.3% of the country’s land area [21, 22]. It straddles the Qinghai-Tibet Plateau, the Loess Plateau and the North China Plain, and eventually merges into the Pacific Ocean. The upstream of the Yellow River is an important runoff-producing area with an average annual runoff of 24.87 billion m^3^, accounting for more than 40% of the whole river. There are seven large reservoirs between the section from Longyangxia to Lanzhou because of the large slope. Among them, LYX and LJX reservoirs have multi-year regulation and annual regulation capacity respectively. These two reservoirs are operated jointly to meet the water demand for power generation and irrigation in Northwest China. But as climate change intensifies, it will have a certain impact on the operation of cascade reservoirs [23]. The geographical location and the spatial distribution of meteorological and hydrological stations in this study are shown in Fig 1.

Previous works found that the climate models CanESM2, CNRM-CM5, MIROC5 and BNU-ESM (they are referred to as CAN, CN5, MI5 and BNU) are more adaptable in the Yellow River Basin, so these four climate models were selected in this study [24, 25]. This study collected meteorological data from 26 basic meteorological stations around and within the basin from 1965 to 2010, as well as daily runoff data from Tangnaihai and Xiaochuan hydrological stations. The future daily meteorological data is from four climate models for the future period (2020–2050) and detailed introduction is shown in Table 1. Seven predictors hur, rhs, sfcWind, tas, va, ua and wap were selected from the NCEP reanalysis and GCM data.

**Table 1:** GCM climate model selection and research area grid selection.

GCMs	Originating Group(s)	Lon	Lat	RCPs	Abbreviation	
CanESM2	Canadian Centre for Climate Modelling and Analysis	95°~105°	32°~37°	RCP2.6/RCP4.5/RCP8.5		CAN
CNRM-CM5	Centre National Recherches Meteorologiques	95°~105°	32°~37°	RCP2.6/RCP4.5/RCP8.5		CN5
MIROC5	Institute of atmospheric and marine research (University of Tokyo)	95°~105°	32°~37°	RCP2.6/RCP4.5/RCP8.5		MI5
BNU-ESM	Beijing Normal University	95°~105°	32°~37°	RCP2.6/RCP4.5/RCP8.5		BNU

Meteorological and hydrological data were obtained from the National Weather Sharing Service Network and the hydrologic data year book respectively. Meanwhile, the historical meteorological data ((S1 Data), the hydrological data (S2 Data) and the four global climate model (GCMs) data (S3 Data) in the manuscript can be downloaded at URLs https://figshare.com/s/74aa686aeda578124fb5 (DOI:10.6084/m9.figshare.19314413). The code of the SIMHYD-SNOW model is developed based on MATLAB platform, which can be downloaded at URLs https://figshare.com/s/b186b63ad33ae9a04e92 (DOI:10.6084/m9.figshare.19314428) and publicly available to anyone.

In addition, inflow above LYX reservoir (TNH) is measured from Tangnaihai station. Inflow between Longyangxia to Liujiaxia (XC) is added by the outflow of Longyangxia Reservoir and the interval inflow.

## 3. Methodology

### 3.1. Nonparametric Mann-Kendall mutation test method

The Mann-Kendall mutation test (M-K) is widely used to check the mutability of meteorological and hydrological data sequences [26, 27]. The *UF* and *UB* curves should be drawn in the same coordinate system at first. When *UF*>0, it means that the time series has a rising trend and vice versa. If the two curves exceed the critical value of U_*α*/2_, it is considered increasing or decreasing significantly. If the two curves intersect inside the critical interval, the crossover point corresponds to the mutation year of the sequence. It is worth noting that most of the meteorological and hydrological data series will be converted from daily-scale into annual-scale first, and then use this method for mutation test.

### 3.2. Statistical downscaling model (SDSM)

The SDSM model includes two major aspects. One is to establish a multiple regression model to find the statistical relationship between the meteorological data measured from ground stations and the NCEP atmospheric circulation factors. The other one is using the SDSM downscale model driven by the GCMs data to establish statistical relationship between the atmospheric circulation factors and the measured meteorological data such as precipitation, daily minimum temperature, daily maximum temperature, wind speed, etc. Then downscaling the future climate scenarios generated by the GCMs model into different meteorological stations of the basin to generate precipitation, daily minimum temperature, daily maximum temperature, and wind speed data sequences [28].

### 3.3. SIMHYD-SNOW model

The SIMHYD hydrological model is a lumped conceptual daily rainfall-runoff model, which has been widely used to simulate the rainfall-runoff process throughout Australia. The model structure and parameters are detailed in the reference published by Chiew [29]. This hydrological model has been widely used in the Yellow River Basin and achieved better application results [30, 31].

**Fig 2:**
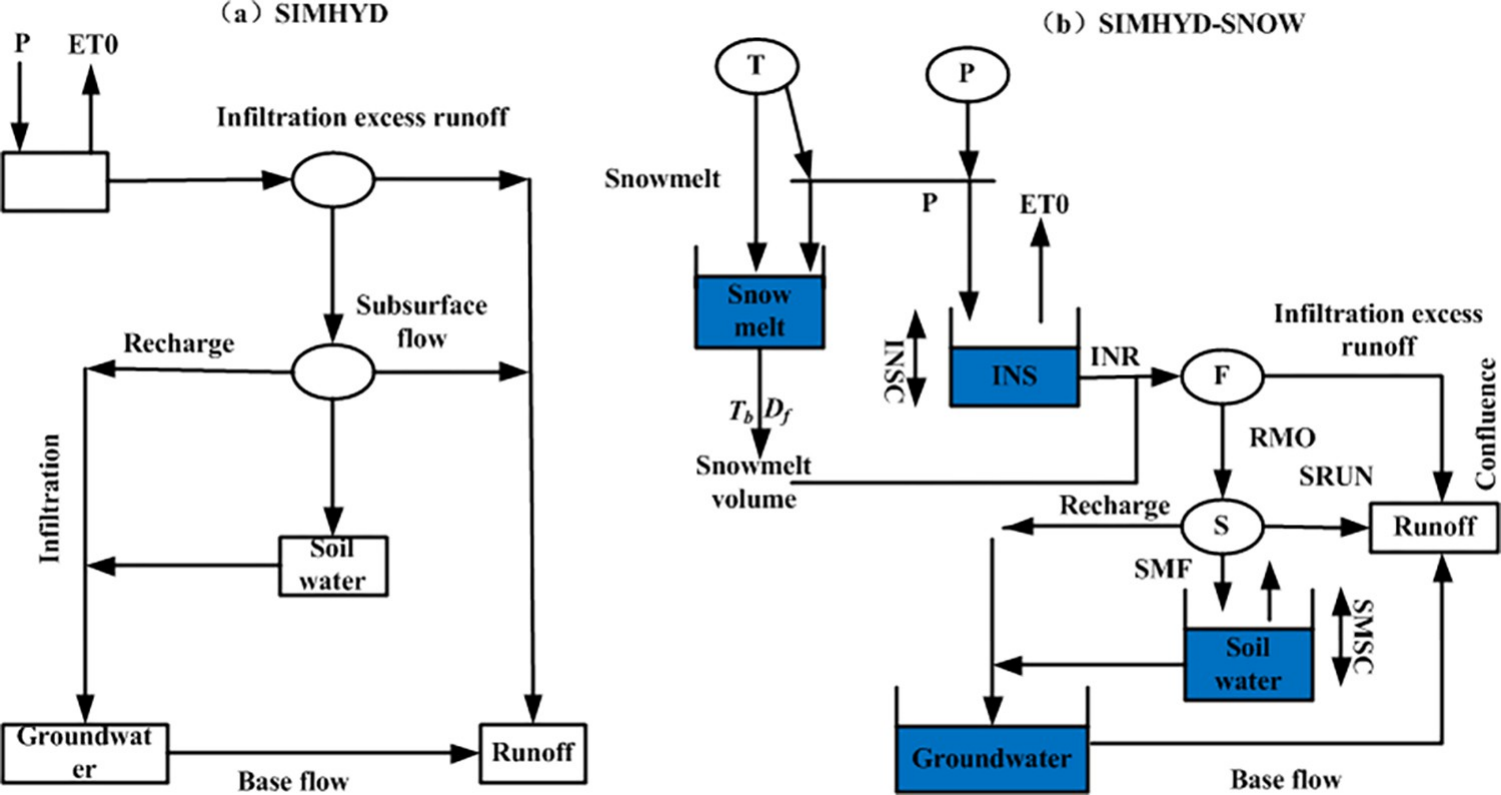
The schematic diagrams of the traditional SIMHYD model and the improved SIMHYD-SNOW model.

The study area in this paper is located in a high-latitude and high-altitude region with low temperature all the year round. The effective precipitation is integrated by snowmelt and rainfall. In order to fully consider this climate feature, a new lumped model called SIMHYD-SMOW was proposed by adding the degree-day factor method that considering the snowmelt process to the traditional SIMHYD model. The precipitation is separated into rainfall and snowfall based on the snowmelt temperature threshold. The degree-day factor method is used to calculate the daily snowmelt volume. The effective precipitation added by the rainfall and the snowmelt volume was used in the runoff generation process in the basin. The schematic diagrams of the traditional SIMHYD model and the improved SIMHYD-SNOW model are shown in Fig 2.

In this paper, the data was divided into warm-up period (1986), calibration period (1987–2000) and validation period (2001–2010). The PSO algorithm was applied to calibrate model parameters with Nash efficiency coefficient (*NSE*) as the objective function [32], and the *KGE*, *RMSE* and *R*^2^ were selected as model accuracy evaluation indexes [33–35]. Table 2 represents the physical significance and calibration parameter values of SIMHYD-SNOW hydrological model parameters.

**Table 2:** Parameters of SIMHYD-SNOW hydrological model.

Parameter	Physical description	Parameter optimal value	
		SIMHYD-SNOW	SIMHYD
INS	Plant retention and storage capacity/mm	0.28	0.10
COEFF	Maximum infiltration loss /mm	56.44	178.70
SQ	Infiltration loss index	76.50	4.28
SMSC	Free water storage capacity /mm	4.07	46.24
SUB	Soil flow coefficient	0.44	0.00
CRAK	Groundwater recharge coefficient	0.29	0.42
K	Base-flow regression coefficient	0.01	0.03
Tb	Snowmelt temperature threshold /°C	3.59	/
DDF	Degree-day factor (mm/°C)	0.53	/

### 3.4. Optimal dispatching model for maximum power generation of cascade reservoirs on the upper Yellow River

The operation effect of the cascade reservoirs on the upper reaches of the Yellow River is dominated by the Longyangxia Reservoir and the Liujiaxia Reservoir.

The Longyangxia Reservoir is a multi-year regulating reservoir with an effective storage capacity of 19.35 billion m^3^, a dead water level of 2530 m, a normal high water level of 2600 m, a flood limit water level of 2594 m, and a designed annual power generation value of 59.24 kW. Liujiaxia Reservoir is an annual regulating reservoir with an effective storage capacity of 2.03 billion m^3^, a dead water level of 1,696 m, a normal high water level of 1735 m, a flood limit water level of 1726 m, and a designed annual power generation value of 57.6 kW.

Taking Longyangxia Reservoir and Liujiaxia Reservoir as research objects to establish a maximum power generation model, the calculation formula is as follows.


$$MaxE=\sum_{i=1}^{N}\sum_{t=1}^{T}\left(N(i,t)\times \Delta T\right)$$ (1)

Where N(i,t) is the average output of the ith power station during t period(MW), ΔT is length of the operating period(month), *E* is the power production generated by the cascade reservoirs (kW), *N* represents the total number of period, and *T* represents the number of reservoirs.

The constraint conditions satisfied by the maximum power generation model are as follows.

Constraints on water balance: $V_{i,t+1}=V_{i,t}+(QI_{i,t}+Qb_{i,t}-q_{i,t})\Delta T$ Constraints on reservoir water level: $H_{\min}(i,t)\leq H_{i,t}\leq H_{\max}(i,t)$ Constraints on reservoir discharge: $q_{\min}(i,t)\leq q_{i,t}\leq q_{\max}(i,t)$ Constraints on output of power station: $N_{\min}(i,t)\leq N_{i,t}\leq N_{\max}(i,t)$ Where *V*_*i*, *t*_, *V*_*i*, *t+1*_ represent the initial capacity and the terminal capacity of the ith reservoir respectively (m^3^), $QI_{i,t}$、 $Qb_{i,t}$ and $q_{i,t}$, are the inflow, interval flow, and outflow of the ith reservoir during the t period respectively (m^3^/s), $H_{\min}(i,t)$、 $H_{\max}(i,t)$ are the upper and lower limits of the water level of the ith power station during the *t* period(m),$H_{i,t}$ is the water level of the ith power station during the *t* period (m), $q_{\min}(i,t)$ and $q_{\max}(i,t)$ represent the upper and lower limits of the outflow of the ith power station during the t period respectively (m^3^/s), $N_{\min}(i,t)$ and $N_{\max}(i,t)$ are the installed capacity and minimum output of the ith power station during the t period (MW), $N_{i,t}$ is the output for the output of the ith power station during the t period (MW).

## 4. Results and analysis

### 4.1. Mutations in runoff from important control sections of the river basin

**Fig 3:**
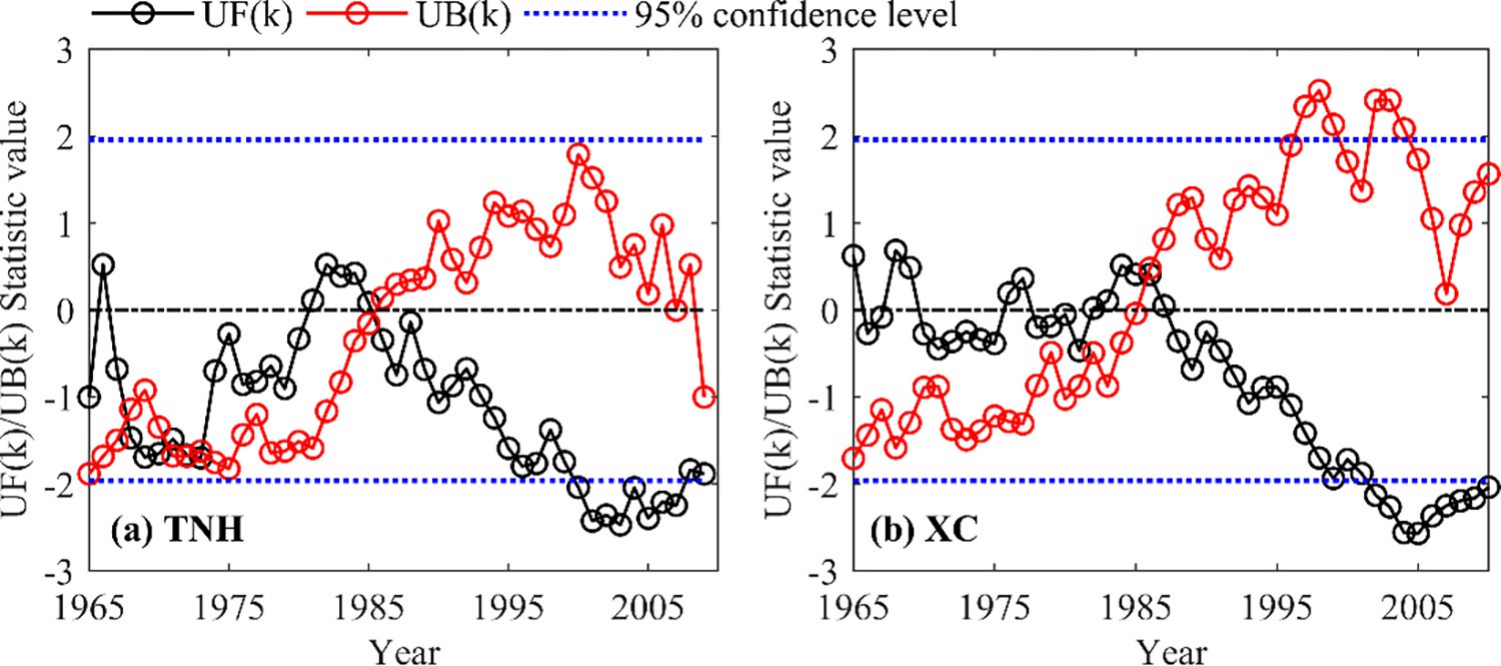
Mutations in runoff sequence at TNH and XC hydrological stations from 1965 to 2010.

Fig 3 shows the results of the annual runoff mutations of Tangnaihai (TNH) and Xiaochuan (XC) hydrological stations from 1965 to 2010 tested by the Mann-Kendall method.

We found that the abrupt change years of annual runoff series of TNH and XC hydrological stations are different. Under Tangnaihai hydrological station, there are multiple intersections of *UF* and *UB* lines, that is, the possible mutation years are 1967, 1973 and 1985 respectively. Among them, under the first two mutation points, the *UF* line fluctuates up and down, and when it is close to 1985, the *UF* line is greater than 0, that is, the runoff shows an increasing trend. Then, after 1985, the *UF* line gradually decreased and passed the 95% significance test in 2000, this means that the annual runoff of TNH and XC hydrological stations shows a decreasing trend. Therefore, we believe that 1985 should be the mutation point of Tangnaihai hydrological station due to climate change and strong human activities. Meanwhile, under the Xiaochuan hydrological station, there is only one mutation point, that is, 1985 is the mutation year. This may be attributed to the rapid economic and social development after the sudden change in China and the water consumption of human beings has increased significantly [30].

### 4.2. Accuracy evaluation of downscaling SDSM model on TNH and XC sub-catchments

**Fig 4:**
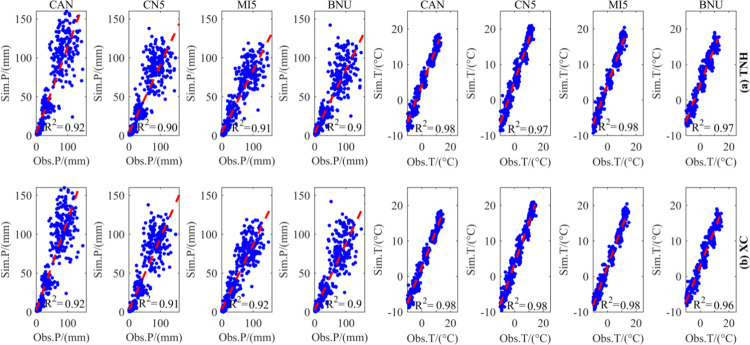
Accuracy evaluation of precipitation and temperature in the TNH and XC sub-basins from 1965 to 2005.

Fig 4 shows the adaptation evaluation of the monthly precipitation and average temperature measured in the TNH and XC sub-basins from 1965 to 2005 and those simulated by the SDSM model. It can be found that the precipitation and temperature elements of the four climate models (CAN, CN5, MI5 and BNU) obtained by the SDSM method have good correlation with the measured values. The correlation between precipitation is slightly lower than that of temperature but both of them can reach the required accuracy, i.e., the correlation between simulated precipitation and measured precipitation of the former is greater than 0.90, and that of the latter is greater than 0.97. Meanwhile, previous works said that when the correlation between simulated and measured values of climate factors is greater than 0.50, the fitting accuracy is better [16]. At the same time, the simulation accuracy of the TNH sub-basin under the CAN climate model is higher than that of other climate models.

In addition, the correlation between simulated precipitation and measured precipitation in the TNH sub-basin for four climate models (CAN, CN5, MI5 and BNU) is 0.92, 0.90, 0.91 and 0.90, respectively. Similarly, the correlation between simulated precipitation and measured precipitation in the XC sub-basin for four climate models (CAN, CN5, MI5 and BNU) is 0.92, 0.91, 0.92 and 0.90, respectively. Meanwhile, the simulated values of light rain to moderate rain events have relatively good correlation with measured values, while the simulated heavy rain events have poor correlation with measured values. In general, CAN climate model is the best in simulating precipitation and temperature elements, MI5 is the second, and BNU is the worst.

### 4.3. Temporal and spatial changes of future meteorological data under GCMs and RCPs models

#### (1) Temporal variation characteristics

**Fig 5:**
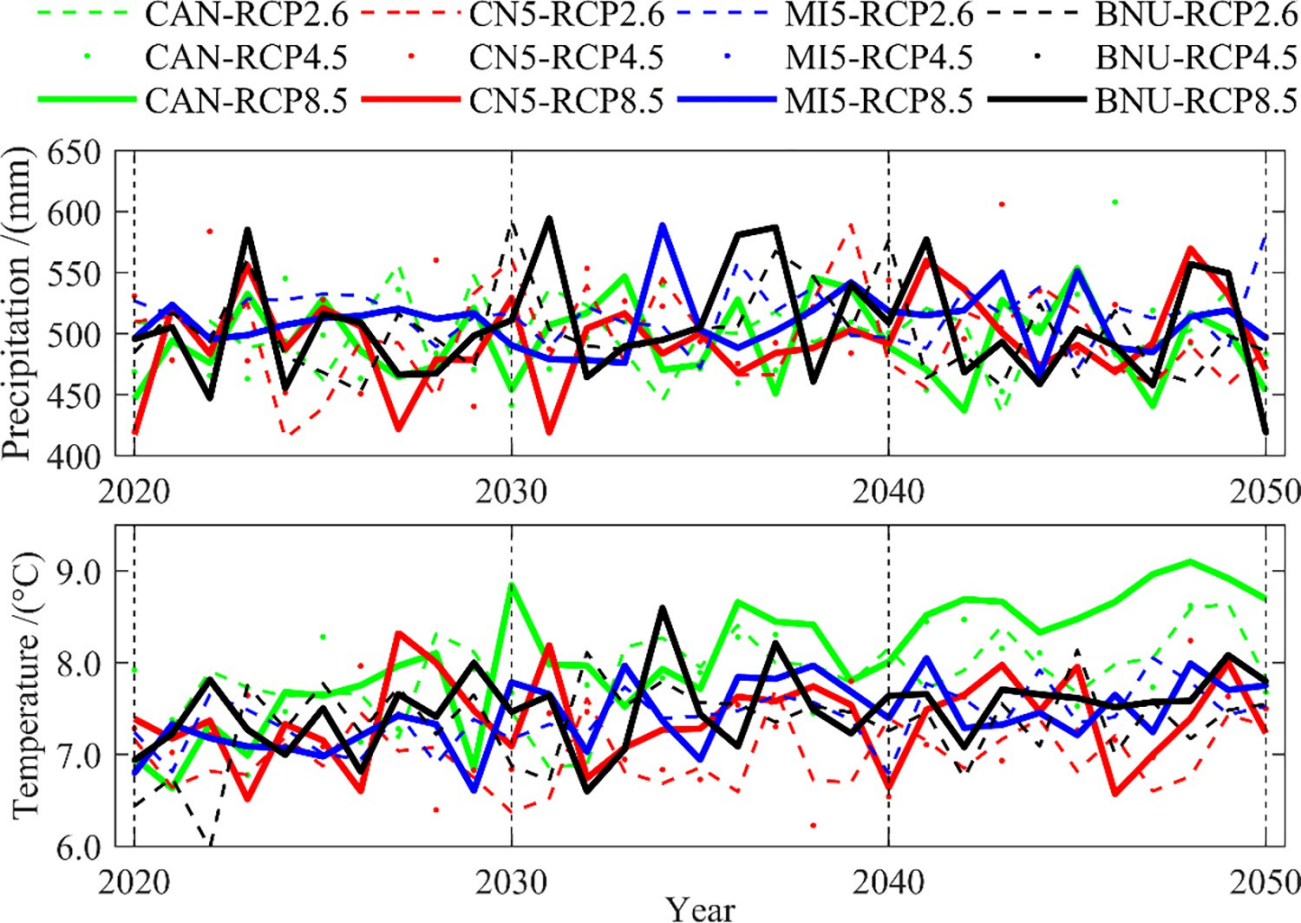
Temporal variation of precipitation and temperature in 2020–2050.

Fig 5 shows the results of temporal changes of precipitation and temperature data in the future period (2020–2050). We can found that, (1) Under different generations (2021–2030, 2031–2040 and 2041–2050), there is a slight upward trend of fluctuations of the precipitation and temperature predicted under different climate change scenarios by four climate models (CAN, CN5, MI5 and BNU). The differences are obvious under different RCPs, for example, during the period of 2041–2050, the temperature increases was largest under the RCP8.5 and the smallest under RCP2.6. Previous works found that with the increase of CO_2_ emission concentration (i.e. from RCP2.6 to RCP8.5), the long-term temperature increases significantly, and there is great uncertainty [16, 18]. (2) The difference in precipitation between the three climate change scenarios under the four climate models is small. The multi-year average precipitation is around 500 mm and different climate change scenarios have less impact on precipitation. (3) The annual average temperature predicted under the CAN climate model is higher than that of other climate models.

The temperature difference is large among different generations. The annual average temperature is 7.80°C, 7.23°C, 7.50°C and 7.96°C under the CAN, CN5, MI5 and BNU climate model respectively, which means that the uncertainty of the climate model has a greater impact on the assessment of temperature.

#### (2) Spatial variation characteristics

**Fig 6:**
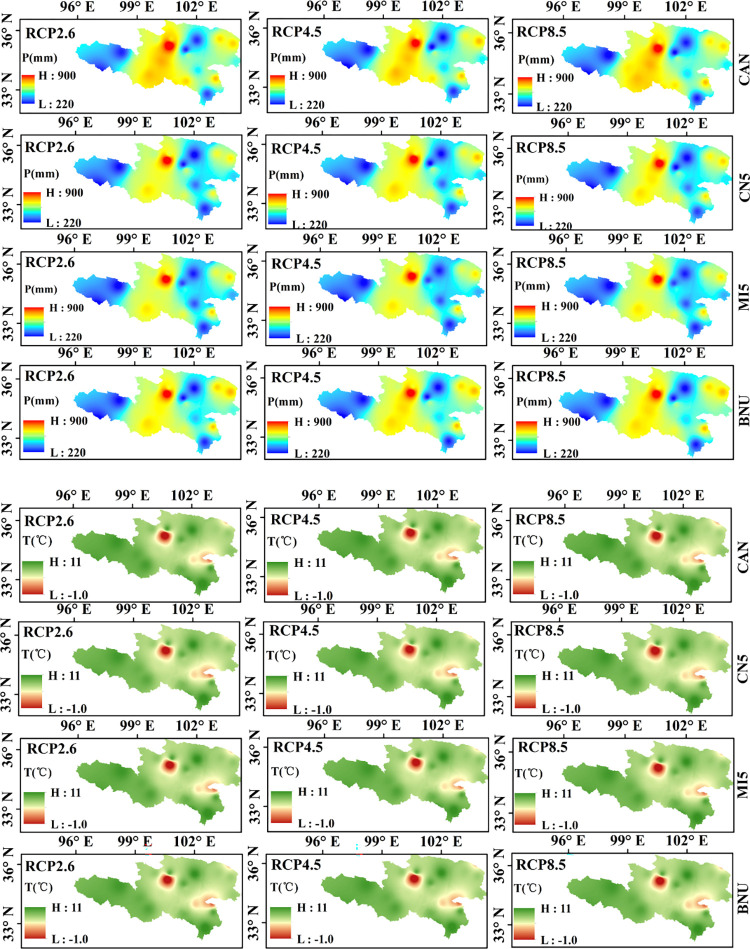
Spatial variation of precipitation (a) and temperature (b) in 2020–2050. (a) Precipitation (b) Temperature.

Fig 6 shows the spatial distribution characteristics of precipitation and temperature in the future period (2020–2050). It can be seen from the figure that, first, the spatial changes of precipitation and temperature predicted by the four climate models (CAN, CN5, MI5 and BNU) under different scenarios are basically the similar. From west to east, the precipitation will increase first and then decrease in the future. The changes are same under different RCPs and GCMs.

Meanwhile, the annual average precipitation is the smallest (between 213.37 mm to 246.97 mm) in the northwest of the basin. But near the TNH hydrological station, the annual average precipitation is as high as 900 mm, which means that this area is prone to extreme flooding events. Similar to the spatial distribution of precipitation, the annual average temperature of the basin is gradually increasing from the northeast to the southwest under different RCPs and GCMs. The temperature in the source area of the Yellow River with high altitude is relatively high, which will accelerate the melting of glaciers and snow.

In general, the uncertainty of climate models and different scenarios have little impact on the spatial distribution of precipitation and temperature in the upper Yellow River Basin, but there are differences in magnitude.

### 4.4. Adaptability evaluation of SIMHYD-SNOW model and future runoff changes

**Fig 7:**
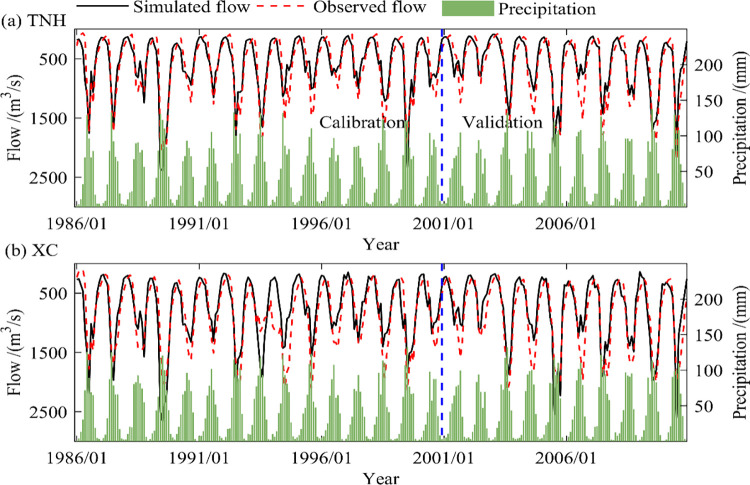
Measured and simulated runoff during the calibration and validation period.

Table 3 and Fig 7 are the evaluation results of the TNH and XC sub-basins. It can be seen from Table 3 that the SIMHYD-SNOW model constructed in this paper has better adaptability in the two sub-basins (TNH and XC) at the regular rate and validation period. The *KGE* during the calibration and validation period are both greater than 0.78 and R^2^ is greater than 0.80. The *KGE*, *RMSE* and *R*^2^ are 0.89, 206.68, 0.93 and 0.78, 211.91, 0.93 during the calibration and verification period respectively of the TNH sub-basin. The *KGE*, *RMSE* and *R*^2^ are 0.80, 286.60, 0.82 and 0.81, 260.32, 0.90 during the calibration and verification period respectively of the XC sub-basin.

**Table 3:** Accuracy evaluation results of the SIMHYD-SNOW model.

Sub-basins	Calibration			Validation		
	*KGE*	*RMSE*	*R* ^2^	*KGE*	*RMSE*	*R* ^2^
TNH	0.89	206.68	0.93	0.78	211.91	0.93
XC	0.80	286.60	0.82	0.81	260.32	0.90

Fig 7 shows that for the two sub-basins (TNH and XC), the precipitation process and the runoff process are in good agreement (more precipitation along with large runoff). The simulation process of the SIMHYD-SNOW model is similar to the measured hydrological process, but the runoff in the dry year is overestimated. This is mainly due to the small runoff of the river basin during that period. The Yellow River Basin has large agricultural water requirements and large water withdrawals for domestic production, which resulting in low flow. The SIMHYD-SNOW model constructed in this paper did not consider the impact of human activities [20].

**Fig 8:**
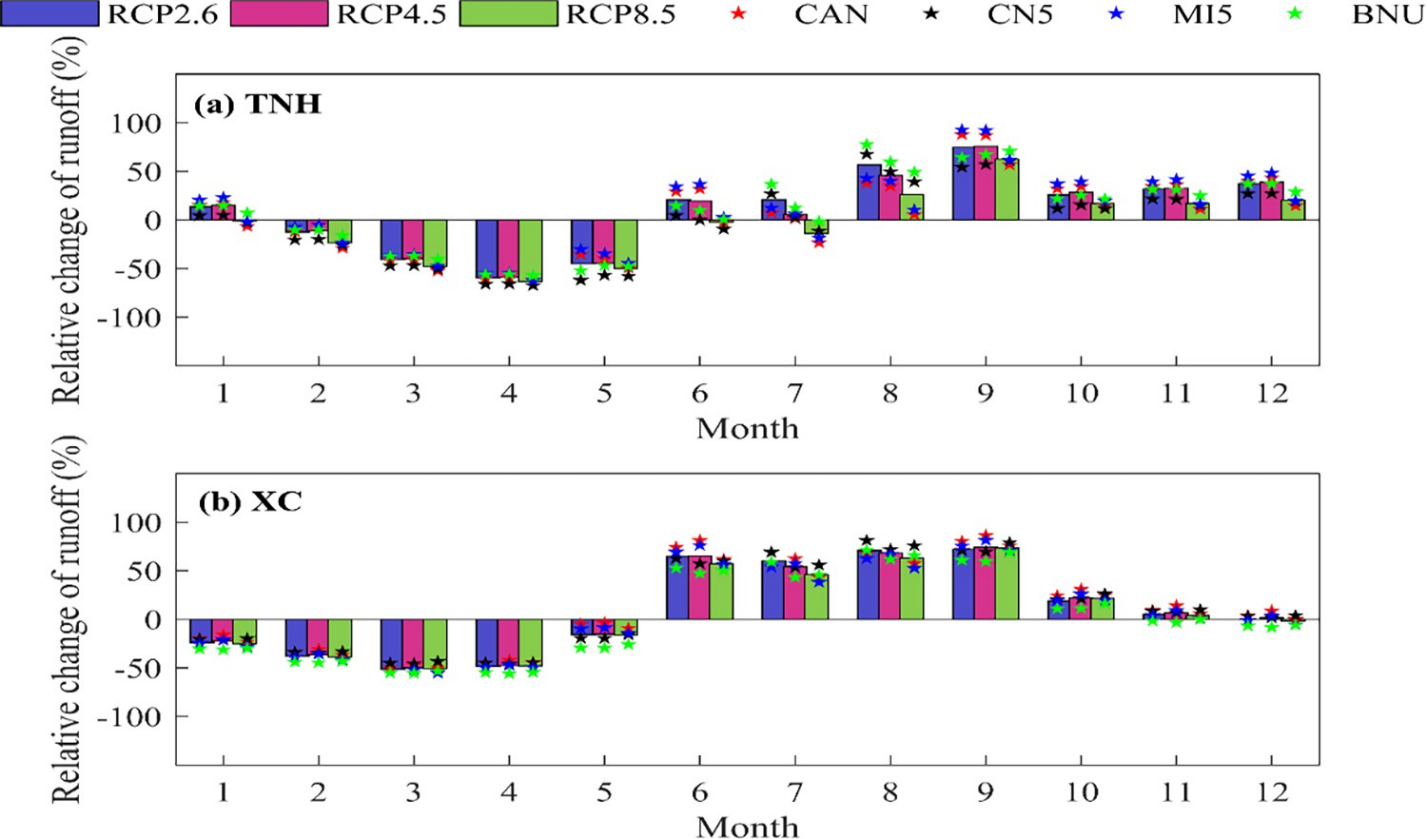
Future percentage change of runoff (bar graph represents the ensemble average of four GCMs under different RCPs).

Fig 8 shows the relative changes of runoff during 2020 to 2050 of the two sub-basins (TNH and XC) on a monthly scale. It can be seen that compared to the baseline period (1986–2010), the relative changing trend of the inner-annual runoff is the same of the two sub-basins. The future runoff will decrease from January to May and increase from June to December. Especially during the flood season(from June to September), there is a significant increase, which means the contradiction between water supply and demand will be alleviated during flood season but will be intensified during the non-flood season.

Taking TNH sub-basin as an example, the relative decrease in runoff will be greater than 50% from March to April, while in June, August, and September, the relative increase in runoff will be greater than 50%. There are differences under different climate change scenarios. For example, in June, the relative change in runoff is 58.63% under RCP2.6 while only 32.46% under the RCP8.5.

In addition, comparing the relative change of runoff under three climate change scenarios (RCP2.6, RCP4.5 and RCP8.5), it can be seen that the increase of runoff under BNU climate model is the largest, MI5 is the second, and CN5 is the smallest in the flood period. Meanwhile, in non-flood period, the decrease of runoff under RCP8.5 is the largest, while the reduction under RCP2.6 is small. But in flood period, the increase of runoff under RCP2.6 is the largest, while the increase is the smallest under RCP8.5. This may be attributed to the smaller future temperature increase in the former, resulting in lower transpiration of soil and vegetation and higher effective precipitation.

### 4.5. Impact of climate change on power generation of cascade reservoirs

#### (1) Total power generation

**Fig 9:**
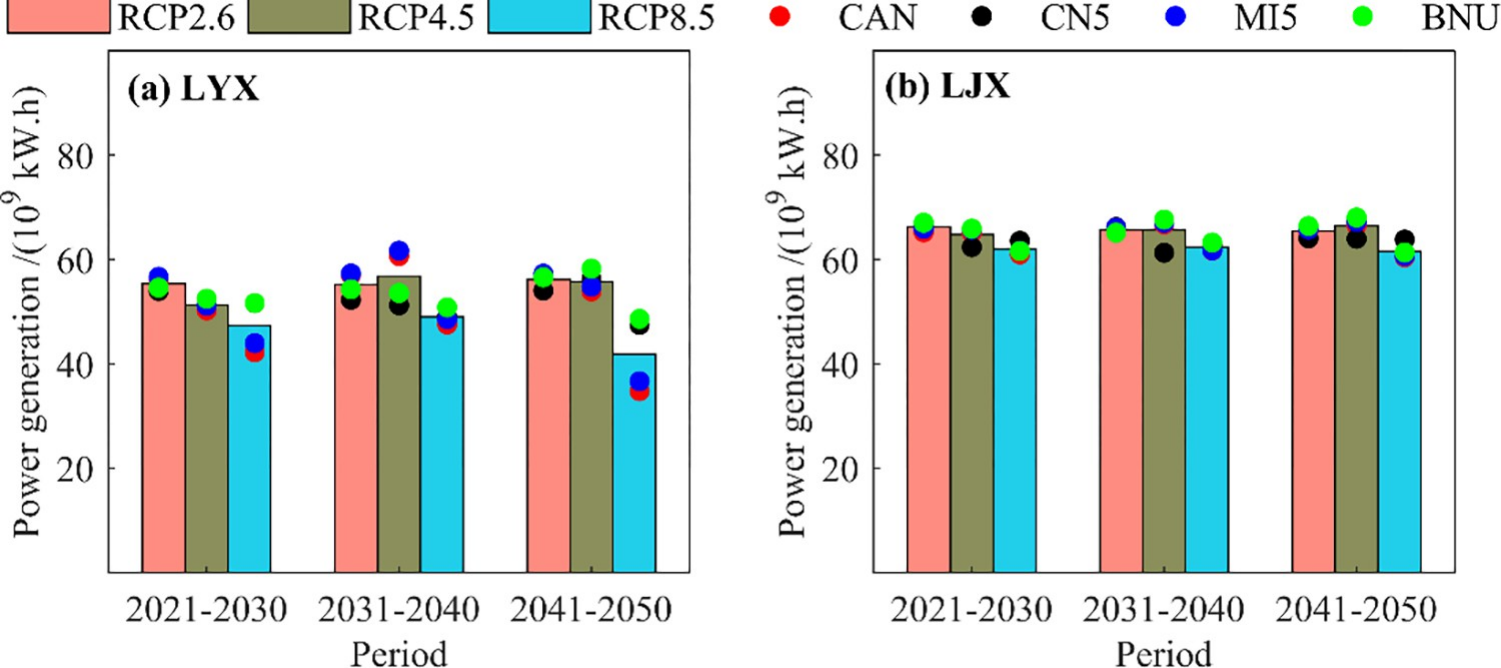
Total power production of LYX and LJX reservoirs under different climate models.

Fig 9 shows the impact of climate change on the total power generation of Longyangxia and Liujiaxia in different generations. It can be seen from the figure: (1) under different climate scenarios (RCPs), there are certain differences in the multi-year average power generation of Liujiaxia reservoir is greater than the Longyangxia reservoir. Meanwhile, the hydropower production of Longyangxia reservoir has changed greatly and that of Liujiaxia reservoir is relatively stable. (2) Different climate change scenarios (RCPs) greatly affect the power generation of the reservoirs, and as the concentration of CO_2_ emissions increasing, the average annual hydropower production has shown a decreasing trend. (3) The hydropower generation has gradually decreased along time, and the impact of climate change on the Liujiaxia reservoir is particularly obvious. (4) The average annual hydropower production of Liujiaxia reservoir under RCP2.6, RCP4.5 and RCP8.5 conditions in 2021–2030, 2031–2040 and 2041–2050 are 55.05×10^9^ kW·h, 50.86×10^9^ kW·h, 46.94×10^9^ kW·h, 54.65×10^9^ kW·h, 55.95×10^9^ kW·h, 48.29×10^9^ kW·h, 55.40×10^9^ kW·h, 55.06×10^9^ kW·h and 41.14×10^9^ kW·h respectively.

In general, climate change has a greater impact on the total power generation of cascade reservoirs in the upper Yellow River, and the power generation significantly reduced under the RCP8.5 scenario.

#### (2) Inner-annual power generation

**Fig 10:**
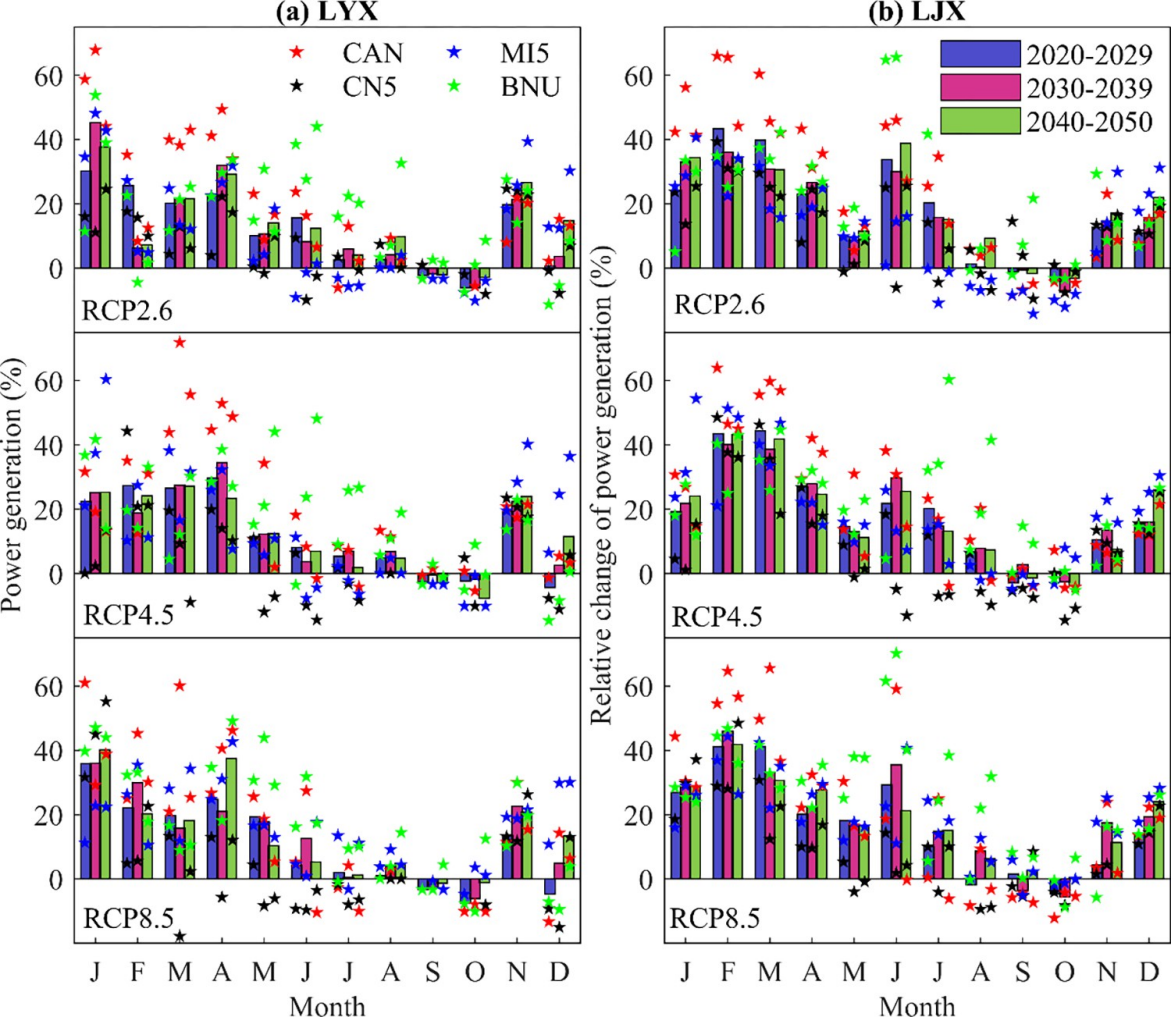
Inner-annual power generation of LYX and LJX reservoirs under different climate models.

In order to assess the impact of climate change on the future power generation of cascade reservoirs within a year, this study took the operating results from 1986 to 2010 as the baseline, and divided the future 2021–2050 into three periods (2021–2030, 2031–2040) And 2041–2050). The percentage change rate was used to reveal the impact of climate change on the inner-annual power generation. Fig 10 shows the impact of climate change on the inner-annual power generation of cascade reservoirs in the future. We found that under different RCPs, the relative changes of future power generation of Longyangxia reservoir and Liujiaxia reservoir are different. Due to the reduction of inflow in non-flood period, in order to meet the requirements of downstream river ecology and agricultural water supply, Longyangxia reservoir and Liujiaxia reservoir increase discharge, resulting in large power generation in this period. However, in the flood period, the water inflow will increase in the future, in order to meet the flood control requirements, Longyangxia reservoir and Liujiaxia reservoir operate at low head, which reduces the optimization of power generation. In detail, future hydropower generation will increase during non-flood period (January-June and November-December).

Meanwhile, the increase rate of future hydropower production in LJX reservoir is significantly higher than LYS reservoir and with the increase of RCPs, the power generation will decrease.

In addition, comparing the monthly scale of power generation during different periods in the future (2020–2029, 2030–2039 and 2040–2050), it can be found that the power generation under RCP8.5 model is significantly reduced during flood season(July-September), and the reduction is greater than other climate change scenarios.

In general, the uncertainty of the climate models affect the inner-annual power generation of the cascade reservoirs. It will increase during the non-flood period while decrease during the flood period, which reflects that under the dual influence of climate change and human activities, the temporal and spatial distribution law of water resources has changed. Reservoir operation is an important way to realize the temporal and spatial redistribution of water resources and solve the contradiction between supply and demand of water resources.

## 5. Discussion

This study comprehensive considered the characteristics of the alpine climate zone in the upper reaches of the Yellow River and the glacier and snow melting process accelerated by climate warming. An improved lumped hydrological model named SIMHYD-SNOW was proposed with the snow melting process. However, due to the watershed was generalized as a homogeneous unit and fails to reflect the spatial heterogeneity, there may be a certain degree of structural uncertainty [29, 32–34]. The five lumped hydrological models (XAJ, HBV, SIMHYD, IHACERS and GR4J) were used to explore the transferability of hydrological models under changing environments and found that the five models show similar performance under stable conditions. At the same time, many studies have found that the uncertainty of climate models has a greater impact on the evaluation of precipitation and temperature elements [16, 24, 25].

Although the CAN, CN5 MI5 and BNU climate models have small impact on the temporal and spatial changes of precipitation and temperature, but have greater changes in future runoff and cascade power generation. Using multiple GCMs can avoid potential unexpected errors of a single model, but different GCMs have different regional applicability. Improper combination of GCMs may affect the prediction results, and the computational complexity of multiple GCMs data is much larger, making climate prediction using multiple GCMs more difficult than using a single model. At the same time, past research has shown that a single model suitable for the study area can also achieve good results. On the basis of summarizing previous studies, we found that the CAN, CN5 MI5 and BNU models are more adaptable in the Yellow River Basin and have better applicability than most other models [35, 36]. Meanwhile, this study aims to reveal the impact of climate change on runoff and power generation of cascade reservoirs in alpine climate regions. It does not consider the uncertainty of model structures and climate models.

In addition, the CMIP6 climate model data set will be used to drive different hydrological models with snowmelt process so as to explore the impact of model uncertainty on runoff changes and power generation processes in the future, thereby improving the forecasting accuracy of inflow and reducing the risk of flood disasters.

## 6. Conclusions

Climate warming has accelerated the snowmelt process in the alpine region of the upper Yellow River, which has changed the temporal and spatial distribution pattern of water resources in this area. This study proposes a hydrological model considering the snowmelt-runoff process named SIMHYD-SNOW and a joint operation model of cascade reservoirs to explore the impact of climate change on hydropower generation. The main conclusions are as follows, (1) the abrupt runoff sequences in the upper Yellow River were concentrated in the 1990s, and it decreased significantly after 2000. These may be closely related to industrial and agricultural water use. (2) Under GCMs and RCPs, the runoff will increase during flood period but decrease during non-flood period in the future. (3) Compared with the climate change scenarios of RCP2.6 and RCP4.5, the hydropower generation in different periods of the upper Yellow River under RCP8.5 has been significantly reduced, especially in 2041–2050.

## Supporting information

S1 Data (XLSX)

S2 Data (XLSX)

S3 Data (XLSX)

S1 File (M)

S2 File (M)
